# Disruption of Ant-Aphid Mutualism in Canopy Enhances the Abundance of Beetles on the Forest Floor

**DOI:** 10.1371/journal.pone.0035468

**Published:** 2012-04-25

**Authors:** Shuang Zhang, Yuxin Zhang, Keming Ma

**Affiliations:** State Key Laboratory of Urban and Regional Ecology, Research Center for Eco-Environmental Sciences, Chinese Academy of Sciences, Beijing, People's Republic of China; University of Utah, United States of America

## Abstract

Ant-aphid mutualism is known to play a key role in the structure of the arthropod community in the tree canopy, but its possible ecological effects for the forest floor are unknown. We hypothesized that aphids in the canopy can increase the abundance of ants on the forest floor, thus intensifying the impacts of ants on other arthropods on the forest floor. We tested this hypothesis in a deciduous temperate forest in Beijing, China. We excluded the aphid-tending ants *Lasius fuliginosus* from the canopy using plots of varying sizes, and monitored the change in the abundance of ants and other arthropods on the forest floor in the treated and control plots. We also surveyed the abundance of ants and other arthropods on the forest floor to explore the relationships between ants and other arthropods in the field. Through a three-year experimental study, we found that the exclusion of ants from the canopy significantly decreased the abundance of ants on the forest floor, but increased the abundance of beetles, although the effect was only significant in the large ant-exclusion plot (80*60 m). The field survey showed that the abundance of both beetles and spiders was negatively related to the abundance of ants. These results suggest that aphids located in the tree canopy have indirect negative effects on beetles by enhancing the ant abundance on the forest floor. Considering that most of the beetles in our study are important predators, the ant-aphid mutualism can have further trophic cascading effects on the forest floor food web.

## Introduction

In addition to predation and competition, mutualism is an important factor in shaping community structure and diversity [1], [2]. Ant-aphid mutualism is a common mutualistic interaction in the field, where ants feed on the honeydew excreted by aphids and in return protect those insects from predators and parasitoids [3], [4]. Aphids lead to a high abundance of ants in the tree canopy [5], and aphid-tending ants have important ecological impacts for both the host plant and other related insect species on the plant [6], [7], [8].

Many honeydew-feeding ants are ground rather than canopy dwelling, such as the ant species in the genera *Formica* and *Lasius* [9], [10], [11], [12], [13] as well as some invasive ants in the genera *Anoplolepis*, *Solenopsis*, *Linepithema* [14], [15], [16]. Plant-based food (such as extrafloral nectar and honeydew excreted by aphids) may be a key factor in creating high densities of ants in the field [17], [18]. For example, the addition of artificial carbohydrates to the forest floor can largely enhance the foraging activity of ants and intensify their interactions with other arthropods [19]. Therefore, aphids may enhance the abundance of ants in the canopy as well as on the forest floor. However, the magnitude of the dependency of ants on aphids is rarely studied, with most existing studies only evaluating the impacts of ants on aphids [3].

Because of their ubiquity and aggressiveness, ants are likely to influence all other arthropod groups on the forest floor, either directly or indirectly [20]. Ants can depress or enhance the diversity or abundance of other arthropod groups on the forest floor [21], [22]. Recent studies have highlighted the importance of ants in structuring the arthropod community in terrestrial ecosystems [19], [23], [24], [25], suggesting that variations in ant abundance can lead to corresponding changes in the arthropod community on the forest floor. Therefore, if aphids in the canopy can change the abundance of ants on the forest floor, the ecological effects of ants on other arthropods can also be changed. Most related studies, however, only focus on the impacts of the ant-aphid interaction on plants [6], [7], [11], [14], [26], [27], [28], [29], [30], and we know little about the possible impacts of this mutualism extend to the forest floor.

In this study, we hypothesized that the breakdown of the ant-aphid mutualism in the canopy could decrease the abundance of ants on the forest floor due to a lack of food resources, and increase the abundance of other ground arthropods. To test this hypothesis, we raised the following questions: 1) Does the exclusion of ants from the canopy impact the abundance of ants and other arthropods on the forest floor, and 2) is there a relationship between the abundance of ants and other arthropods in the field?

## Methods

### Ethics statement

No specific permits were required for the described field studies. The location was not privately owned or protected in any way, and the study did not involve endangered or protected species.

### Study site

The study area is located in the Beijing Forest Ecosystem Research Station (30°57′29 N, 115°25′33 E, altitude 1,200–1,400 m), a member of the Chinese Ecological Research Network (CERN), about 100 km northwest of Beijing City, China. This area typically has a warm temperate continental monsoon climate with average annual precipitation of 500–650 mm. The mean annual temperature is 5–10°C. It is an oak (*Quercus liaotungensis*) dominated, 80-year-old secondary forest with a few birches (*Betula spp*.), maples (*Acer mono.*), and shrubs (e.g., *Prunus spp.*, *Vitex negundo* var. *hetertophylla*).

### Impacts of excluding ants from the canopy on the abundance of ants and other arthropods on the forest floor

We conducted this experiment during three consecutive growing seasons (2009, 2010, and 2011) of the oak tree *Q. liaotungensis*, which is the dominant tree species in the study area [31]. We selected a slope in a small watershed to conduct the experiment. We chose this area because the ant *Lasius fuliginosus* was the only active ant species with high abundance in the pitfall trap sampling in this area. *L. fuliginosus* is a typical honeydew-feeding ant that has mutualistic relationships with some aphid species [32]. In the study area, *L. fuliginosus* was attracted by aphids *Lachnus tropicalis* and *Tuberculatus sp.* in the canopy and *Stomaphis japonica* on the trunk of *Q. liaotungensis*. The aphid was the key factor attracting ants in the canopy of *Q. liaotungensis* in the study site [33].

**Figure 1:**
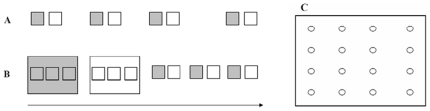
The experimental design in 2009 (A), 2010, and 2011 (B) and the sampling sites in each 20*20 m plot (C). Grey represents treated plots and white represents control plots. The size of the large plots in 2010 was 80*60 m. The arrows represent the spatial arrangement of the treated and control plots, where along the arrow (from left to right), the basic lines of ant abundances decreased, but the basic lines of beetle and spider abundances increased.

In 2009, we set up four pairs of plots (20*20 m) with a distance of at least 50 m between the adjacent pairs. For each pair, the left plot was set as the aphid exclusion plot and the other as the control plot (Fig. 1A), with a distance of more than 15 m between the treated and control plots. We used this experimental design because, based on our observation, the abundance of ants decreased and other arthropod abundances increased from left to right in the study site (as the arrow shows in Fig. 1). This was a conservative experimental design to test our hypothesis, as before the experiment, the ant abundance in the treated plots was higher than in the control plots. If the ant abundance in the treated plots was significantly lower than in the control plots after the treatment, we could confirm that the treatment significantly impacted the ant abundance. The tree density, leaf area index (LAI), and canopy coverage in the treated and control plots were not significantly different (Table S1).

Based on the results from 2009, we set up two large plots (80*60 m) in 2010 and 2011 to evaluate the impacts of the exclusion area on the results. The large plots included two pairs of the small plots used in 2009 (Fig. 1B). Three pairs of small plots (20*20 m) were also set up (two pairs were used in 2009) for comparison with the large plots. For the large plots, the left was set as the treatment and the right as the control, as in the conservative experimental design in 2009.

In April of each year in the study (before the growing season), an adhesive ring was smeared around the trunk (about 1 m above the ground, and 5 cm in width) on all trees in the treatment plot to impede the access of ants to aphids on the canopy. The adhesive was made of a polymer resin mixture (Beijing Nonghaha S & T CO. LTD) and was nontoxic, harmless to plants, and non-attractive to insects. The adhesive was re-smeared every two months during the growing season until the end of the study. Any bridges that could allow ants to climb onto trees were cut off throughout the study.

Pitfall traps were used for arthropod sampling. This method is a sampling technique extensively used to sample surface foraging invertebrates such as ants, beetles, and spiders [25], [34], [35]. According to a systematic sampling method, we set up 16 traps in each 20*20 m plot, with a 5 m interval between adjacent traps (Fig. 1C). We collected samples only in the three small plots in the middle area of the large plot in order to reduce any possible edge effect (Fig. 1B). Starting at the end of May in 2009, 2010, and 2011, we sampled the abundance of ground arthropods each month. For each trap, a cup (diameter = 7.9 cm, depth = 9.7 cm) with 50 ml of alcoholic solution (5% in concentration) was buried under the ground. Two days after the traps were set, we retrieved the cups and samples were taken back to the lab for taxonomy. We then classified the arthropods as ants, spiders, beetles, centipedes, millipedes, caterpillars, and so on, and counted the number of individual arthropods in each family. The species of ants, spiders, and beetles were identified as accurately as possible.

### Relationships between the abundance of ants and other arthropods in the field

We also conducted a field survey to evaluate the relationship between the spatial distribution pattern of ants and other arthropods, such as beetles and spiders, in the field. We carried out this work monthly in May and June 2010. We set up three parallel transects along a slope (T1, T2, T3, with a width of 10 m) in the study site that were 130 m, 80 m, and 140 m in length, respectively (with distance more than 200 m from each other). The transect length was limited by the distance from the bottom to the top of the slope. We divided each transect into 10*10 m plots, so there were 13, 8, and 14 corresponding plots. In each plot, three traps (the same as mentioned above) were set according to a regular triangle design (the trap as the vertex), with 5 m between each trap. The sampling method was the same as that used in the experimental study.

T1 and T3 were at the left and right of the experimental study area, respectively (the direction is shown by the arrow in Fig. 1), but both were far from the nearby experimental plots (>50 m). T2 crossed the left center part of the experimental study area, but we avoided sampling in the experimental plot. Therefore, the samplings in the three transects were unlikely to be affected by the experimental study.

### Data analysis

A mixed effects model for data with spatial autocorrelation was used to analyze our data [36]. In this model, the data collected in the same plot were considered as repeated measures with spatial autocorrelation. To conduct the analysis, each data point was associated with its coordinates in a plot. For each trap in a plot, the data of different months was averaged to correct the temporal autocorrelation. In the mixed effects model, the model of the variogram was selected by matching the three theoretical variograms (Gaussian, exponential, and spherical) with the data. Data for plots of different sizes were analyzed separately.

For each year, the effect of the aphid-exclusion treatment on the abundances of ants, beetles, spiders, and predators on the forest floor was analyzed. Then, the data from different years were averaged to evaluate the overall effects of the ant-exclusion treatment on the forest floor throughout our study period.

The mixed effects model was also used with the field survey data to evaluate the variation in the abundances of ants and other arthropods on the forest floor in the three transects. The data of the two months were averaged for each trap. Data collected in the same plot were considered as spatial repeated measures with autocorrelation. We also analyzed the relationship between the abundances of ants and other arthropods at the plot scale in the three transects. In this analysis, data collected in each plot were averaged. The mean values for each variable in a plot were transformed by ln(n+1). Then, a generalized linear regression was used to evaluate the relationship between the abundances of ants and other arthropods. All the analyses were conducted using the SAS statistical package (SAS Institute Inc.).

## Results

### Impacts of aphid-exclusion on the abundance of ants and other arthropods on the forest floor

During the 3-year study, 176,994 ants (95.6%), 2,844 beetles (1.5%), 3,356 spiders (1.8%), and 1,956 others (1.1%) were collected. The non-ant captures were mainly beetles and spiders, and the effects of ants on those two groups were analyzed and discussed explicitly. In the group of ants, 99.9% belonged to *L. fuliginosus*, with 0.07% belonging to *Formica fusca* Linnaeus and 0.02% belonging to *Formica sinenis*. For beetles, 85.7% belonged to the Carabidae family. For spiders, 41.2% belonged to Opiliones, 28.4% belonged to Gnapphosidae, and 10.8% belonged to Linyphiidae.

The ant-exclusion treatment had no significant effects on the tested variables in the small plots during any of the three consecutive years (2009–2011; all *p*>0.05). In the large plots, the abundance of ants was reduced by the treatment in both 2010 and 2011, although in 2010, this effect was only marginally significant (F_1,4_ = 6.78, *p* = 0.0598). In 2010, the abundance of beetles was 35.6% higher in the large treated plot than in the control plot, but the difference was insignificant (F_1,4_ = 4.97, *p* = 0.0900). In 2011, beetle abundance in the large treated plot was 80.7% higher than that in the control plot (F_1,4_ = 17.76, *p* = 0.0140). The abundance of spiders was not significantly affected by the treatment in either of the two years (for both years, *p*>0.05). However, as compared with the control plot, the abundance of spiders was 60.4% higher in the large treated plots in 2011 (see detailed information about the effects of treatment in each year in Table S2 and Fig. S1).

**Figure 2:**
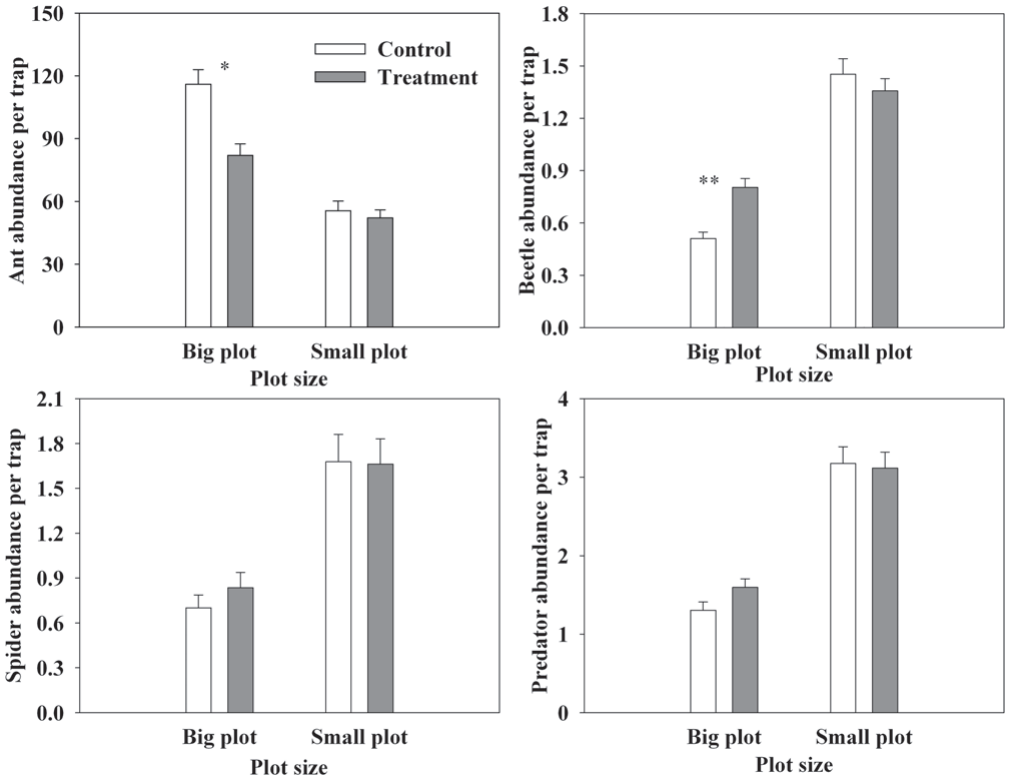
The impacts of excluding ants from the canopy on the abundance of ground arthropods (Mean, SE, * represents *p*<0.05, ** represents *p*<0.01).

Throughout the study period, the abundances of ants and beetles were significantly affected by the treatment in the large plots. However, in the small plots, none of the tested variables were significantly affected by the treatment (Table 1, Fig. 2). In general, the ant abundance in the large treated plots was 27.4% lower than in the control plot. The beetle abundance was 58.8% higher in the large treated plot than in the control plot (Table 1, Fig. 2). The abundance of spiders in the large treated plot was 20.3% higher than in the control plot, but the difference was insignificant (Table 1, Fig. 2).

**Table 1:** The effects of the ant-exclusion treatment from the canopy on the abundances of ants, beetles, spiders and predators on forest floor.

Variable	Plot size	F value	df	P
Ants	Small	0.41	1,8	0.5411
	Big	10.9	1,4	**0.0299**
Beetles	Small	0.29	1,8	0.6043
	Big	21.25	1,4	**0.0100**
Spiders	Small	0.00	1,8	0.9675
	Big	0.53	1,4	0.5067
Predators	Small	0.01	1,18	0.9089
	Big	2.12	1,4	0.2194

### The impacts of ant exclusion on predators

With the exception of ants, 76.9% of the collected samples were predators, including spiders (42.4%), predatory beetles (beetles belong to Carabidae; 28.3%), and centipedes (6.3%). Therefore, we evaluated the impact of ant exclusion on non-ant predators. In the small plots, the treatment had no significant effect on the abundance of predators in the 3-year study (Table 1). When the data of 2010 and 2011 were averaged, the abundance of predators was 22.7% higher in the large treated plot than in the control plot, but the difference was insignificant (Table 1, Fig. 2). When the data from different years was analyzed separately, we found that the treatment significantly increased the abundance of predators in the large plots in 2011 (F_1, 4_ = 7.59, *p* = 0.0510, Table S2, Fig. S1).

### Relationships between the abundance of ants and other arthropods in the field

Along the three transects, 26,813 individual arthropods were collected. The most abundant group was ants (96.4%), followed by beetles (1.6%), spiders (0.9%), and others such as centipedes, millipedes, caterpillars, and so on (1.1%). In the ant group, 99.2% belonged to *L. fuliginosus*.

**Figure 3:**
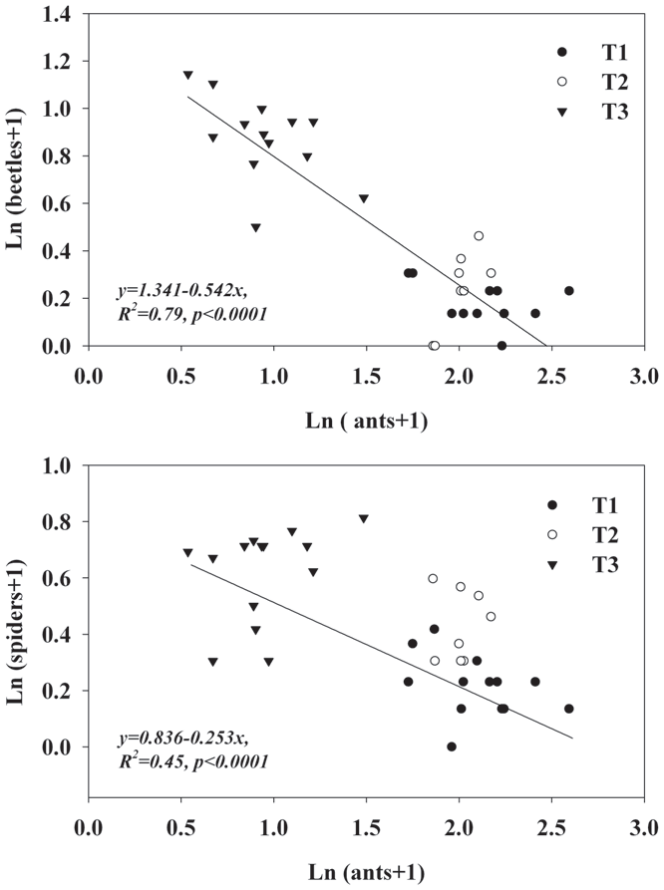
The relationship between the abundance of ants and beetles, and ants and spiders in the three transects (n = 35).

The three transects varied largely in the abundance of ants, beetles, and spiders (Fig. 3; for ants, F_2, 32_ = 16.40, *p*<0.0001, for beetles, F_2, 32_ = 40.29, *p*<0.0001, for spiders, F_2, 32_ = 27.91, *p*<0.0001). The abundances of beetles and spiders were higher at transects with a lower abundance of ants. At the plot scale in the three transects, both the abundances of beetles and spiders were negatively related to the abundance of ants (Fig. 3).

## Discussion

Many studies have found that aphid-tending ants have important impacts on plants and other insects that live on plants [6], [7], [8], suggesting that aphids can shape the arthropod community by attracting ants. Through a 3-year experimental study and a field survey, we confirmed that aphids also enhance the abundance of ants on the forest floor, thus leading to a decrease in the abundance of beetles on the ground. Our findings suggest that the ant-aphid mutualism in the canopy also has crucial ecological effects for the forest floor.

We confirmed that the loss of access to aphids can significantly suppress the activity of aphid-tending ants on the forest floor. Most studies on the ant-aphid interaction have only evaluated the impacts of ants on aphids [3], [4], [37]. Recently, several studies have focused on the effects of hemiptera on ants [17], [19], [38], [39]. We found that after the loss of aphids in the canopy, the ant abundance on the forest floor decreased by 27.4% in the large treated plot. This suggests that aphid presence in the canopy plays an important role in mediating the activity of ants on the forest floor. It is possible that the exclusion of ants from the canopy can make other food resources less available for ants, but according to our observation, *L. fuliginosus* rarely carried other insects down the tree, suggesting that their main interest in the canopy is aphids. *L. fuliginosus* feed not only on honeydew in the canopy, but also on trunks along with the aphid *Stomaphis japonica*, and probably from the roots of the host tree [40]. This is one possible reason why the abundance of ants can stay at a high level in the large treated plots.

The size of the ant-exclusion area is also an important factor in our results. For *L. fuliginosus*, the foraging radius is about 30 m [41], so the 20*20 m exclusion plots in the current study may be too small to show the impacts of aphids on ants on the forest floor. It is intuitive to assume that the larger the exclusion area, the more likely the ants will avoid that area, resulting in a strong decline in ant abundance. Even large areas of ant-exclusion from the canopy (e.g., at the local scale) are necessary to fully evaluate the impacts of the ant-aphid mutualism on the forest floor.

Our study suggests that the aphid is an important factor shaping the structure of the arthropod community on the forest floor. The ant exclusion treatment decreased the abundance of ants on the forest floor, and the abundances of beetles significantly increased. In the field, a clear pattern appears where the abundances of beetles and spiders are strongly related to the abundance of ants (Fig. 3), suggesting negative effects of ants on the abundances of beetles and spiders. Sites with a high abundance of ants may not be suitable habitats for other arthropods such as beetles [19]. A previous study found that the abundance of Carabids is largely suppressed by ants [42], and our research supports this argument. We observed that, on the forest floor, beetles were attacked by *L. fuliginosus* when they met. Although the experimental study results suggest a negative effect of ants on spiders, the effect was not significant. In the field survey, we also found that the negative relationship between spiders and ants was much weaker than the relationship between ants and beetles (Fig. 3). Ants and spiders are often competitors [43]; they can even prey on each other [22], which can complicate the outcomes of the ant-spider interaction. In our study site, we observed that spiders belonging to Opiliones often quickly ran away when they met *L. fuliginosus*, and the *L. fuliginous* also seemed to stay away from spiders. Therefore, the possible effect of ants on spiders (e.g., the effects on spider behavior) still needs further investigation. Given that Carabids are generalist predators in the terrestrial ecosystem [44], the cascade effects of those predators on other arthropods on the forest floor should be considered in future studies.

The key weakness of the present study is the pseudoreplication in the large plots [45], which reduced the reliability of our results. Finding comparable large plots with homogenous biotic and abiotic environments (such as the abundances of ants, beetles, spiders, and other arthropods, the size and densities of the focal tree species, canopy coverage, and so on) was quite difficult in the mountain ecosystem of the study area. As we showed in the field survey of the three transects, the spatial variation of arthropod abundance is very large. The abundances of arthropods show clear spatial gradient patterns. For example, in our study area, the paralleled transects T1 and T3 varied about 50 times in the abundance of ants, 19 times in the abundance of beetles, and 6 times in the abundance of spiders (Fig. 3). The spatial gradient pattern of arthropod abundances in our study area also hampers the establishment of comparable nearby large plots. Even with the above weakness, our results are reliable for several reasons. First, along the spatial gradient of the abundance of ants and other arthropods, we adopted a conservative experimental design to evaluate the effects of the ant-exclusion treatment. Second, through the sampling process in 2009, we confirmed that the abundances of ants, beetles, and spiders were homogenous in the sites for which we set up the two large plots in 2010 and 2011. Thus, the two sites are ideal for conducting the large plot ant-exclusion treatment. Compared to the pattern in the small plots, the difference between the two large plots in the abundance of ants and beetles after the treatment is quite clear, suggesting strong effects of the ant-exclusion treatment. Third, as a complement to the ant-exclusion experiment, the field survey results also suggest strong negative effects of ants on beetles in the study area. Based on the above argument, the study is reliable at a certain level and the results are meaningful as a preliminary study on the effects of the ant-aphid mutualism on other arthropods on the forest floor. We suggest that the sampling of aphids on the canopy, ants and other arthropods on the forest floor across a large number of sites may be a useful method to avoid the limitation of setting up large comparable plots in future studies.

In conclusion, we found support for the existence of ecological impacts of the ant-aphid mutualism on the forest floor: this mutualism can enhance the abundance of ants but suppress the abundance of beetles. This study extends our understanding of the ecological impacts of the ant-aphid mutualism from the canopy to the forest floor. Considering the common, widespread occurrence of ant-aphid interactions in a variety of ecosystems [3], it is important to fully evaluate the possible impacts of the interaction beyond plants. Future studies should focus on the impacts of this mutualism on key ecological patterns and processes on the forest floor, such as the arthropod community structure and diversity, and nutrient processes such as C and N cycling. The answers for those questions are crucial for extending our knowledge of the role of ant-aphid interactions in the community and ecosystem.

## Supporting Information

Figure S1 **The effects of ant-exclusion from canopy on the abundances of ants, beetles, spiders and predators on the forest floor (mean, SE).**
(TIF)

Table S1 **The leaf area index (LAI), cover and tree densities in the treated and control plots.**
(DOC)

Table S2 **The effects of ant-exclusion from canopy on the abundances of ants, beetles spiders and predators on the forest floor.**
(DOC)
