# Supplementary material for: Disruption of Ant-Aphid Mutualism in Canopy Enhances the Abundance of Beetles on the Forest Floor
Source: PLoS One. 2012 Apr 25;7(4):e35468. doi: 10.1371/journal.pone.0035468 (PMC3338844; doi:10.1371/journal.pone.0035468)
Supplement: Table S1 — The leaf area index (LAI), cover and tree densities in the treated and control plots. (DOC) [file pone.0035468.s002.doc]

Table S1 The leaf area index (LAI), cover and tree densities in the treated and control plots.

| Year | Variable | Treatment (mean, SE) | Control (mean, SE) | P value |
| --- | --- | --- | --- | --- |
| 2009 (small plots) | Lai | 1.83 (0.09) | 1.75 (0.09) | 0.69 |
|  | Cover | 79.2% (1.5%) | 77.8% (1.5%) | 0.68 |
|  | Tree density | 38.0 (5.5) | 30.5 (4.5) | 0.25 |
| 2010 (large plots) | Lai | 1.63 (0.14) | 1.76 (0.12) | 0.47 |
|  | Cover | 74.0% (3.7%) | 77.5% (2.0%) | 0.67 |
|  | Tree density | 40.33 (6.5) | 36.67 (5.7) | 0.83 |
| 2010 (small plots) | LAI | 1.64 (0.11) | 1.65 (0.12) | 0.49 |
|  | Cover | 75.6% (1.8%) | 75.4% (2.3%) | 0.55 |
|  | Tree density | 33.33 (5.5) | 24.33 (1.5) | 0.13 |
