# Supplementary material for: Disruption of Ant-Aphid Mutualism in Canopy Enhances the Abundance of Beetles on the Forest Floor
Source: PLoS One. 2012 Apr 25;7(4):e35468. doi: 10.1371/journal.pone.0035468 (PMC3338844; doi:10.1371/journal.pone.0035468)
Supplement: Table S2 — The effects of ant-exclusion from canopy on the abundances of ants, beetles spiders and predators on the forest floor. (DOC) [file pone.0035468.s003.doc]

Table S2 The effects of ant-exclusion from canopy on the abundances of ants, beetles spiders and predators on the forest floor

| **Variable** | **Year** | **Plot size** | **F value** | **df** | **P** |
| --- | --- | --- | --- | --- | --- |
| Ants | 2009 | Small | 0.07 | 1.6 | 0.8303 |
|  | 2010 | Big | 6.78 | 1,4 | 0.0598 |
|  | 2010 | Small | 0.77 | 1,4 | 0.4297 |
|  | 2011 | Big | 9.24 | 1,4 | **0.0380** |
|  | 2011 | Small | 0.01 | 1,4 | 0.9457 |
| Beetles | 2009 | Small | 1.20 | 1.6 | 0.3153 |
|  | 2010 | Big | 4.97 | 1,4 | 0.0900 |
|  | 2010 | Small | 0.89 | 1,4 | 0.3993 |
|  | 2011 | Big | 17.76 | 1,4 | **0.0140** |
|  | 2011 | Small | 1.08 | 1,4 | 0.3577 |
| Spiders | 2009 | Small | 0.02 | 1.6 | 0.8812 |
|  | 2010 | Big | 0.44 | 1,4 | 0.5454 |
|  | 2010 | Small | 0.02 | 1,4 | 0.9056 |
|  | 2011 | Big | 3.17 | 1,4 | 0.1496 |
|  | 2011 | Small | 0.01 | 1,4 | 0.9387 |
| Predators | 2009 | Small | 0.10 | 1.6 | 0.7629 |
|  | 2010 | Big | 0.09 | 1,4 | 0.7851 |
|  | 2010 | Small | 0.12 | 1,4 | 0.7469 |
|  | 2011 | Big | 7.59 | 1,4 | **0.0510** |
|  | 2011 | Small | 0.16 | 1,4 | 0.7090 |
